# The great masquerader of malignancy: chronic intestinal pseudo-obstruction

**DOI:** 10.1186/s40364-014-0023-y

**Published:** 2014-12-05

**Authors:** Josephine A Taverna, Hani M Babiker, Seongseok Yun, Maria C Bishop, Sarah Lau-Braunhut, Paul N Meyer, Thomas Enzler

**Affiliations:** Division of Hematology-Oncology, Department of Medicine, Southern Arizona VA Health Care System, 3601 S 6Th Ave, Tucson, AZ 85723 USA; Division of Hematology-Oncology, Department of Medicine, University of Arizona Cancer Center, 1515 N Campbell Ave, Tucson, AZ 85724 USA; University of Arizona College of Medicine, 1501 N. Campbell Ave, Tucson, AZ 85724 USA; Department of Medicine, University of Arizona College of Medicine, 1501 N. Campbell Ave, Rm 6336, Tucson, AZ 85724 USA

**Keywords:** Paraneoplastic chronic intestinal pseudo-obstruction, Anti-Hu antibodies, Myenteric ganglioneuritis, Small cell lung cancer

## Abstract

Paraneoplastic syndromes can precede the initial manifestation and diagnosis of cancer. Paraneoplastic syndromes are a heterogeneous group of disorders caused by mechanisms other than the local presence of tumor cells. These phenomena are mediated by humoral factors secreted by tumor cells or by tumor mediated immune responses. Among paraneoplastic syndromes, chronic intestinal pseudo-obstruction (CIPO) is rare and represents a particularly difficult clinical challenge. Paraneoplastic CIPO is a highly morbid syndrome characterized by impaired gastrointestinal propulsion with symptoms and signs of mechanical bowel obstruction. Clinical outcomes of paraneoplastic CIPO are often deleterious. The current standard of care for the management of CIPO includes supportive treatment with promotility and anti-secretory agents. However, the majority of patients with CIPO eventually require the resection of the non-functioning gut segment. Here, we present a 62-year-old patient with anti-Hu antibody associated paraneoplastic CIPO and underlying small cell lung cancer who underwent treatment with cisplatin and etoposide. Herein, we discuss diagnosis, prognosis, proposed mechanisms, treatment options, and future potential therapeutic strategies of paraneoplastic CIPO.

## Background

Paraneoplastic syndromes are disorders associated with cancer and reflect the interaction between tumor cells, host cells, and cells of the immune system. They are not a direct effect of the underlying malignancy and occur at remote sites from the primary or metastatic lesions [1,2]. Paraneoplastic neurologic syndromes (PNS) are rare among the paraneoplastic syndromes, and less than 1% of cancer patients are affected by PNS [1]. The pathogenesis of PNS is incompletely understood, however it is thought that immunologic factors such as auto-antibodies and T-cell responses against the nervous system, as well as a breakdown of immune tolerance, play an important role [2-4]. Auto-antibody formation is thought to be triggered by an aberrant expression of neuronal antigens by tumor cells [1]. Examples of well-characterized auto-antibodies in conjunction with PNS are type 1 anti-neuronal nuclear (anti-Hu or ANNA1), anti-voltage gated calcium channel, anti-Yo and anti-amphiphysin antibodies (Table 1) [5]. Paraneoplastic CIPO is a rare PNS mainly associated with small cell lung cancer and carcinoid tumor. Similar to other PNS, the pathophysiologic mechanism hypothetically involves auto-antibody mediated inflammation of the nervous system.

**Table 1:** **Antibodies in paraneoplastic neurologic syndromes**

**Antibody**	**Paraneoplastic neurologic syndrome**	**Associated malignancies**
Anti-Hu (ANNA-1)	Encephalomyelitis, cerebellar degeneration, sensory neuronopathy, autonomic dysfunction	SCLC
Anti-Yo (PCA-1)	Cerebellar degeneration	Gynecological, breast
Anti-Ri (ANNA-2)	Cerebellar degeneration, opsoclonus-myoclonus	Gynecological, breast, SCLC
Anti-Tr (DNER)	Cerebellar degeneration	Hodgkin lymphoma
Anti-amphiphysin	Stiff-person syndrome	Breast, lung cancer
Anti-Ma2 (Ta)	Limbic encephalitis	Teratoma, lung cancer
Anti-CRMP5 (CV2)	Encephalomyelitis, peripheral neuropathy	SCLC, thymoma
Anti-recoverin	Retinopathy	SCLC
Anti-VGCC	Lambert-Eaton syndrome	SCLC
Anti-VGKC	Neuromyotonia	Thymoma
		Hodgkin lymphoma
		SCLC
Anti-AChR	Myasthenia gravis	Thymoma
Anti-titin		
Anti-ryanodine		
Anti-JO1	Inflammatory myopathies	Ovarian cancer
Anti-Mi2		Lung cancer
Anti-p155		Gastric cancer
		Non-Hodgkin lymphoma

## Case presentation

**Figure 1:**
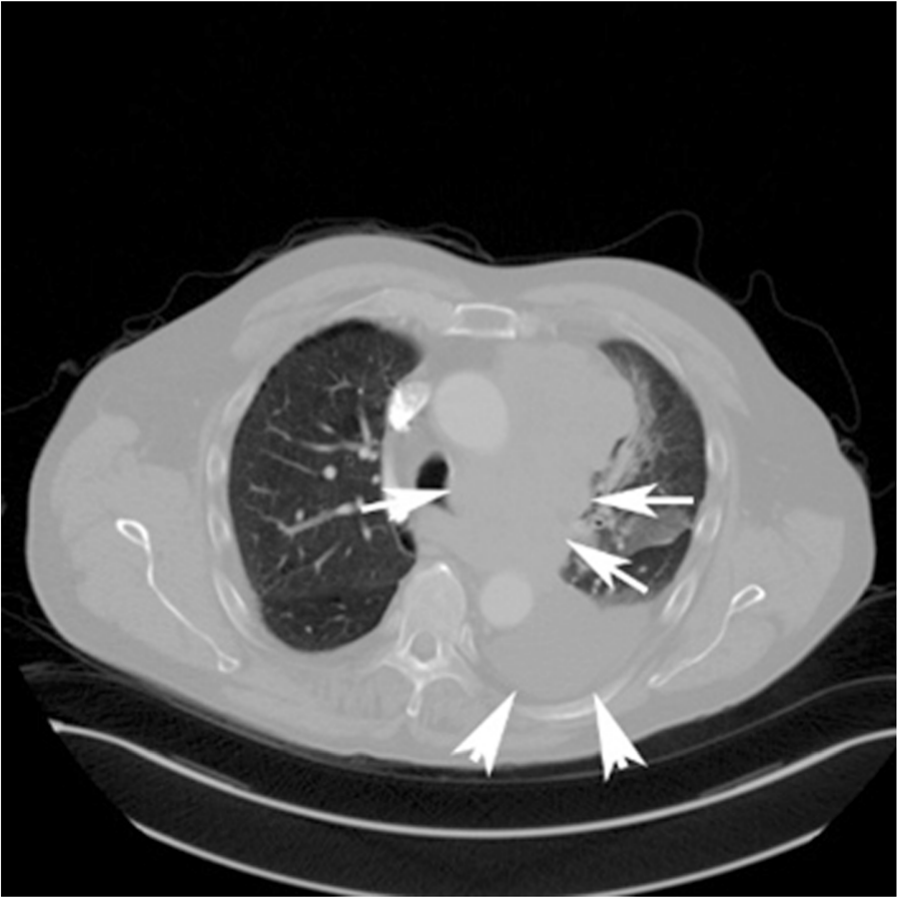
**CT scan of the chest.** CT of the thorax (horizontal section) showing a large mass measuring 4.6 × 12.0 × 8.1 cm, encasing the left pulmonary artery and segmental branches (arrows).The mass also abutts the left mainstem bronchus with partial encasement. Moderate-sized left pleural effusion (arrow heads).

**Figure 2:**
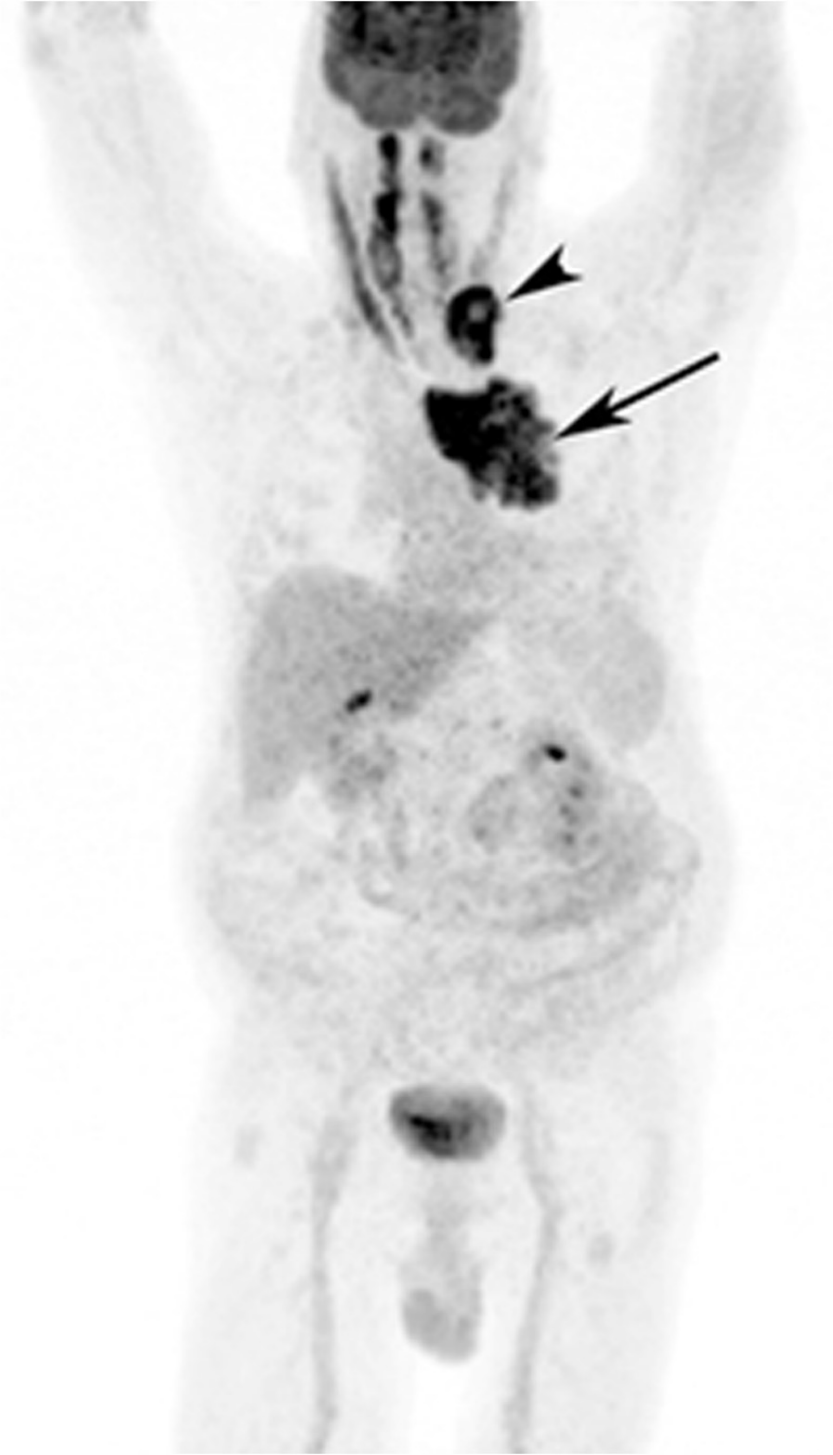
**Whole body PET scan using 18 F-FDG.** PET scan shows a left upper thorax tumor mass (arrow) and metastases to left supraclavicular lymph nodes (arrow head).

**Figure 3:**
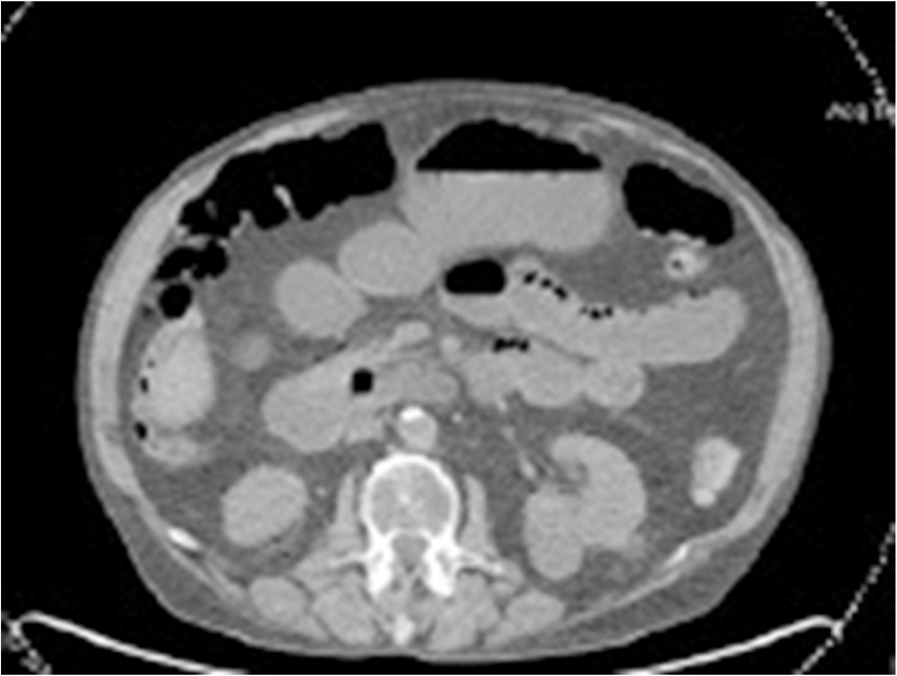
**CT scan of the abdomen.** CT scan of the abdomen showing a distention of small bowel loops with several air-fluid levels.

**Figure 4:**
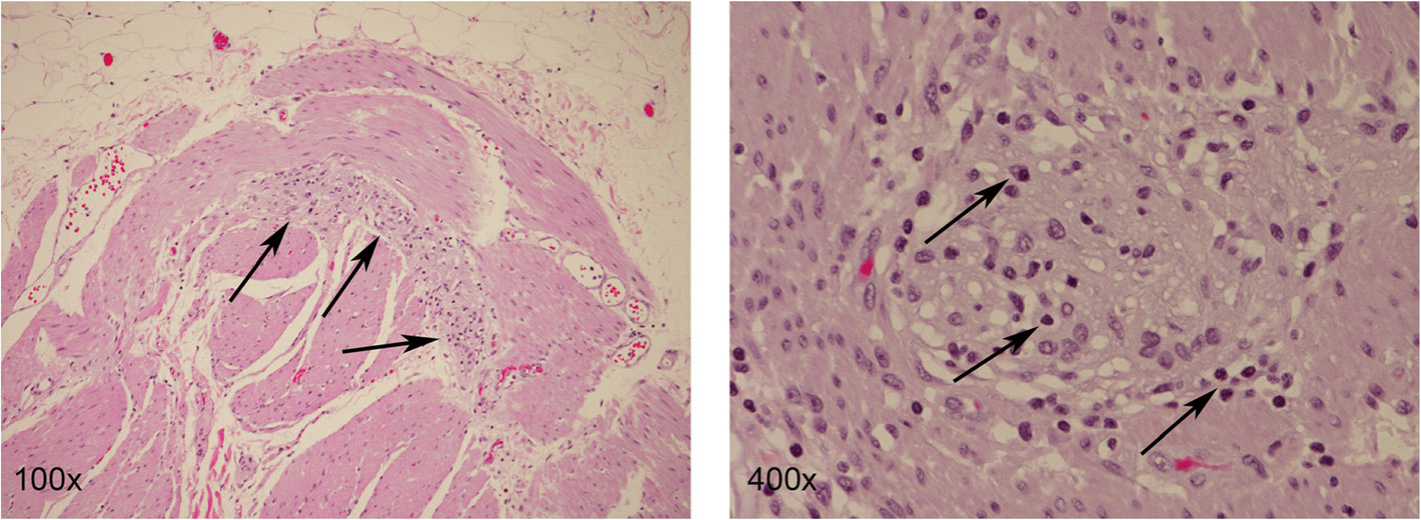
**H & E stains of resected sample of the small intestine.** The myenteric plexi show an increased lymphoid infiltrate (100x, arrows), with a few plexi having a rather dense lymphoplasmacytic infiltrate (400x, arrows) consistent with myenteric ganglioneuritis. Magnification as indicated.

A 62-year-old gentleman with chronic obstructive pulmonary disease (COPD) from chronic smoking presented with worsening cough and constipation for three months. Within the last year, the patient was recurrently treated for COPD exacerbations without significant clinical improvement. The patient denied having had fever, pain, nausea, vomiting, night sweats, or weight loss. Vital signs were unremarkable and pertinent findings on physical exam included mild wheezes on both lungs with decreased breath sounds over the left upper lobe, enlarged left supraclavicular lymph nodes, and hypoactive bowel sounds. Laboratory exams revealed a low sodium concentration of 130 mEq/L (normal 136–145 mEq/L), however other results were all within normal range. Computed tomography (CT) and positron emission tomography (PET) scans showed a large left upper lobe fludeoxyglucose (FDG)-avid mass (standardized uptake value (SUV) 14.3) and enlarged left supraclavicular lymph nodes with avid FDG uptake (SUV 4.0) (Figures 1 and 2). Brain magnetic resonance imaging (MRI) revealed no metastatic disease in the brain. Subsequent fine-needle aspiration of the left supraclavicular mass and immunohistochemistry staining confirmed small cell neuroendocrine cancer positive for thyroid transcription factor (TTF-1) and synaptophysin confirming the diagnosis of small cell lung cancer (SCLC). The disease was determined as extensive disease due to the fact that tumor/nodal volume was too large to be encompassed in a tolerable radiation plan. During the hospital course, the patient’s bowel movements further declined despite an aggressive bowel regimen. Ultimately, the patient developed symptoms akin to bowel obstruction and a CT scan revealed small bowel distention with multiple air-fluid levels (Figure 3). Considering possible mechanical bowel obstruction surgery was consulted and the patient underwent small bowel resection of the terminal ileum and cecum. However, no tumorous obstruction was found and histologic examination of the resected sample revealed intense lymphoplasmacytic infiltration consistent with myenteric ganglioneuritis as this is typically found in CIPO (Figure 4). Anti-Hu antibodies were positive with a titer of 1:640. Collectively, the laboratory and pathologic findings were consistent with paraneoplastic CIPO with underlying SCLC.

10 days after surgery the patient underwent a first cycle of chemotherapy with cisplatin (80 mg/m^2^ on day 1 and etoposide (100 mg/m^2^ on day 1–3) without any improvement of his bowel activities despite promotility and anti-secretory agents including intravenous octreotide (200 mcg, three times a day), metoclopramide (10 mg, three times a day), and dexamethasone (20 mg, once a day). He was given parenteral nutrition. The course was complicated by neutropenic sepsis requiring treatment with broad spectrum antibiotics. After successful recovery the patient was given a second cycle of chemotherapy with cisplatin and etoposide. Anti-Hu antibody levels re-checked prior to the second cycle of chemotherapy demonstrated marked improvement with titers down to 1:160 from 1:640. Repeated CT scan of the chest shortly after the second cycle of chemotherapy revealed substantial shrinkage of the primary tumor and the metastases, however bowel function remained absent. Unfortunately, while neutropenic after the second cycle of chemotherapy the patient developed severe sepsis requiring intubation and intensive care medicine. Despite all the efforts the patient died from multi-organ failure.

## Discussion

Paraneoplastic disorders are present in 7-10% of the cancer patients with malignancies, while paraneoplastic neurological syndromes (PNS) are observed only in 0.01% of cancer patients. PNS are mainly associated with lung, breast, ovarian, and stomach cancer [1,6,7]. Limbic encephalitis, opsoclonus-myoclonus, cerebellar degeneration, stiff person syndrome, retinopathy, CIPO, peripheral neuropathy, Lambert-Eaton syndrome, and myasthenia gravis are examples of PNS (Table 1) [7,8]. Neurologic manifestations associated with these diseases are clinically important because it was found that more than 80% of PNS precede the diagnosis of underlying malignancy [9]. Moreover, PNS are thought to be associated with favorable prognosis and better outcome, as suggested in SCLC patients [1,10,11].

CIPO is characterized by signs and symptoms of mechanical bowel obstruction in the absence of an anatomic lesion [12]. CIPO is idiopathic in most cases. Etiologies causing CIPO include neurologic, endocrine, collagen vascular, paraneoplastic, infectious and genetic disorders (Table 2) [12-14]. Histological features of CIPO include myenteric plexus infiltration with plasma cells and lymphocytes associated with axonal and neuronal degeneration [15,16]. Although paraneoplastic CIPO has been reported in connection with several solid tumors, SCLC and carcinoid tumors are by far the most common culprits [17,18]. Auto-antibodies such as anti-Hu that are frequently found to be positive in *paraneoplastic*, but not in *non-paraneoplastic* CIPO, often precede the overt manifestation of the underlying malignancy, rendering these antibodies to be potentially diagnostic and prognostic markers [2,19]. Anti-Hu antibodies are polyclonal complement-fixing immunoglobulins directed against nuclear proteins expressed in the neurons. A hypothesis is that these antibodies, together with the extensive lymphoplasmacytic infiltration, interact with the enteric plexus leading to its malfunction and, finally, to its irreversible damage resulting in un-correctable gut dysmotility [5,20,21]. To date, a relationship between anti-Hu antibody titers and severity of the clinical symptoms could not be demonstrated and convincing data indicating that a decrease in anti-Hu levels correlates with improved CIPO or better prognostic outcome are lacking [22-24]. In addition to CIPO, anti-Hu antibodies are also known to be involved in paraneoplastic CNS dysfunctions such as limbic encephalopathy and cerebellar degeneration as well as other paraneoplastic motor and autonomic neuropathies [20]. In general, anti-Hu antibodies associated with paraneoplastic neurologic conditions are called anti-Hu syndromes [20,25,26].

**Table 2:** **Etiologies of chronic intestinal pseudo-obstruction**

**Etiology**	**Underlying disorders**	**Mechanisms**
Degenerative neuropathy	Shy-Drager syndrome	Altered Ca signaling
	Diabetes mellitus	Mitochondrial dysfunction
	Parkinson’s disease	
Immune mediated	Scleroderma	Antibody mediated neuron or smooth muscle involvement
	Dermatomyositis	
	Systemic lupus erythematosus	
Paraneoplastic	Small cell lung cancer	Antibody mediated inflammatory response and cellular infiltration
	Carcinoid tumor	
Infectious	Chagas disease	Inflammatory change of the enteric nerve system
	Chronic JC virus infection	
Radiation or chemotherapy induced	Post radiation or chemotherapy	Associated with treatments
Genetic	Hirschsprung disease	Associated with genetic abnormality of SOX10
	Mitochondrial encephalopathy with lactic acidosis and stroke like episodes (MELAS)	DNA polymerase γ
		Filamin A
	Myoclonus epilepsy associated with ragged red fibers (MERRF)	L1 cell adhesion molecule
		Thymidine phosphorylase
		PTEN
		RNA binding protein for multiple splicing 2 (RBPMS2)

Treatment of paraneoplastic CIPO is rarely successful [27-29]. Previous reports revealed the effect of prokinetic as well as anti-secretory agents to be very limited [29]. The combination of chemotherapy with plasmapheresis compared to chemotherapy alone also failed to show improvement in clinical outcome in most cases, although anti-Hu antibodies were successfully removed from the circulation [28]. The lack of clinical response despite the removal of the anti-Hu antibodies is thought to be, at least in part, due to irreversible neuronal damage [5]. The persisting absence of bowel function in our patient despite receiving chemotherapy could also be secondary due to Ogilvie’s syndrome triggered by surgical procedure, critical illness and/or chemotherapy. However, given the histological findings of lymphoplasmacytic infiltration of the intestinal plexus and given the disease course, we believed that paraneoplastic CIPO, and not Ogilvie’s syndrome, was most likely the cause of the absent bowel function in our patient [30]. Recently, Badari et al. and Coret et al. reported cases where patients with paraneoplastic CIPO were partially successfully treated with either combination treatment with rituximab (RTX) and cyclophosphamide or RTX alone [8,31]. A hypothesized mechanism in this scenario is that the inhibitory effect of RTX on B-cells prevents them to function as efficient antigen-presenting cells. This may result in a reduction of B-cell-triggered cytotoxic T-cells, which are directed towards anti-Hu antigens in neurons [31].

The mechanisms involved in anti-Hu antibody-associated PNS appeared tightly linked to the development of a partially efficient anti-tumor immune response [9-11].

Future goals to improve outcomes in paraneoplastic CIPO and other PNS may include the development of effective immunotherapies. As mentioned, PNS often develop prior to the diagnosis of cancer and anti-Hu antibodies were found to be highly specific biomarkers for PNS in the setting of SCLC [9,20]. As such, further studies may also explore the question of whether anti-Hu antibodies can serve as reliable markers for the early detection of SCLC presenting with neurologic symptoms. If so, this may lead to prompt treatment and better clinical outcome of SCLC.

## Conclusion

Paraneoplastic CIPO is rare and difficult to treat. Symptomatic treatments including plasmapheresis, prokinetic, and anti-secretory agents have been tried, but without significant benefit. Recent studies, however, have indicated success with single agent and combined regimens with RTX suggesting a role for immunomodulation in the treatment of CIPO. Future studies focusing on understanding the intricate pathophysiology associated with paraneoplastic CIPO and other PNS will hopefully open new horizons in the management of these rare and highly morbid disorders.

## Consent

Written informed consent was obtained from the patient’s next kin for publication of this Case report and any accompanying images. A copy of the written consent is available for review by the Editor-in-Chief of this journal.

## Abbreviations

CIPO: Chronic intestinal pseudo-obstruction
PNS: Paraneoplastic neurologic syndrome
COPD: Chronic obstructive pulmonary disease
PET/CT: Positron emission tomography/computed tomography
SUV: Standardized Uptake values
FDG: Fludeoxyglucose
TTF-1: Thyroid transcription factor
SCLC: Small cell lung cancer
ANNA-1: Anti-neuronal nuclear antibody
PCA-1: Purkinje cell antibody 1
DNER: Delta/Notch-like epidermal growth factor-related receptor
CRMP-5: Collapsin response mediator protein family 5
VGCC: Voltage gated calcium channel
VGKC: Voltage gated potassium channel
AchR: Acetylcholine receptor
JC virus: John Cunningham virus
MELAS: Mitochondrial encephalopathy with lactic acidosis and stroke like episodes
MERRF: Myoclonus epilepsy associated with ragged red fibers
DNA: Deoxyribonucleic acid
SOX: Sry related HMG box
PTEN: Phosphatase and tensin homolog
RBPMS2: RNA binding protein for multiple splicing 2

## References

[1] Tischler M, Schoenfield Y, Schoenfield Y, Gershwin ME (2000). Paraneoplastic Syndromes. Cancer and Autoimmunity.

[2] Ansari J, Nagabhushan N, Syed R, Bomanji J, Bacon CM, Lee SM (2004). Small cell lung cancer associated with anti-Hu paraneoplastic sensory neuropathy and peripheral nerve microvasculitis: case report and literature review. Clin Oncol.

[3] Voltz R, Dalmau J, Posner JB, Rosenfeld MR (1998). T-cell receptor analysis in anti-Hu associated paraneoplastic encephalomyelitis. Neurology.

[4] Sutton I, Winer JB (2002). The immunopathogenesis of paraneoplastic neurological syndromes. Clin Sci.

[5] Senties-Madrid H, Vega-Boada F (2001). Paraneoplastic syndromes associated with anti-Hu antibodies. Isr Med Assoc J.

[6] Molina-Garrido MJ, Guillen-Ponce C, Martinez S, Guirado-Risueno M (2006). Diagnosis and current treatment of neurological paraneoplastic syndromes. Clin Transl Oncol.

[7] Honnorat J, Antoine JC (2007). Paraneoplastic neurological syndromes. Orphanet J Rare Dis.

[8] Badari A, Farolino D, Nasser E, Mehboob S, Crossland D (2012). A novel approach to paraneoplastic intestinal pseudo-obstruction. Support Care Cancer.

[9] Pignolet BS, Gebauer CM, Liblau RS (2013). Immunopathogenesis of paraneoplastic neurological syndromes associated with anti-Hu antibodies: A beneficial antitumor immune response going awry. Oncoimmunology.

[10] Maddison P, Newsom-Davis J, Mills KR, Souhami RL (1999). Favourable prognosis in Lambert-Eaton myasthenic syndrome and small-cell lung carcinoma. Lancet.

[11] Mawhinney E, Gray OM, McVerry F, McDonnell GV: **Paraneoplastic sensorimotor neuropathy associated with regression of small cell lung carcinoma.** *BMJ Case Rep* 2010, **2010**ᅟ. 10.1136/bcr.01.2009.1486 PMC3038026 22802230

[12] De Giorgio R, Sarnelli G, Corinaldesi R, Stanghellini V (2004). Advances in our understanding of the pathology of chronic intestinal pseudo-obstruction. Gut.

[13] Antonucci A, Fronzoni L, Cogliandro L, Cogliandro RF, Caputo C, De Giorgio R, Pallotti F, Barbara G, Corinaldesi R, Stanghellini V (2008). Chronic intestinal pseudo-obstruction. World J Gastroenterol.

[14] Khairullah S, Jasmin R, Yahya F, Cheah TE, Ng CT, Sockalingam S (2013). Chronic intestinal pseudo-obstruction: a rare first manifestation of systemic lupus erythematosus. Lupus.

[15] De Giorgio R, Guerrini S, Barbara G, Stanghellini V, De Ponti F, Corinaldesi R, Moses PL, Sharkey KA, Mawe GM (2004). Inflammatory neuropathies of the enteric nervous system. Gastroenterology.

[16] Di Nardo G, Blandizzi C, Volta U, Colucci R, Stanghellini V, Barbara G, Del Tacca M, Tonini M, Corinaldesi R, De Giorgio R (2008). Review article: molecular, pathological and therapeutic features of human enteric neuropathies. Aliment Pharmacol Ther.

[17] Sodhi N, Camilleri M, Camoriano JK, Low PA, Fealey RD, Perry MC (1989). Autonomic function and motility in intestinal pseudoobstruction caused by paraneoplastic syndrome. Dig Dis Sci.

[18] Lee HR, Lennon VA, Camilleri M, Prather CM (2001). Paraneoplastic gastrointestinal motor dysfunction: clinical and laboratory characteristics. Am J Gastroenterol.

[19] Darnell RB, DeAngelis LM (1993). Regression of small-cell lung carcinoma in patients with paraneoplastic neuronal antibodies. Lancet.

[20] Dalmau J, Graus F, Rosenblum MK, Posner JB (1992). Anti-Hu-associated paraneoplastic encephalomyelitis/sensory neuronopathy. A clinical study of 71 patients. Medicine.

[21] Lennon VA, Sas DF, Busk MF, Scheithauer B, Malagelada JR, Camilleri M, Miller LJ (1991). Enteric neuronal autoantibodies in pseudoobstruction with small-cell lung carcinoma. Gastroenterology.

[22] Llado A, Mannucci P, Carpentier AF, Paris S, Blanco Y, Saiz A, Delattre JY, Graus F (2004). Value of Hu antibody determinations in the follow-up of paraneoplastic neurologic syndromes. Neurology.

[23] Darnell RB, Posner JB (2003). Paraneoplastic syndromes involving the nervous system. N Engl J Med.

[24] Graus F, Dalmou J, Rene R, Tora M, Malats N, Verschuuren JJ, Cardenal F, Vinolas N, Garcia Del Muro J, Vadell C, Mason WP, Rosell R, Posner JB, Real FX (1997). Anti-Hu antibodies in patients with small-cell lung cancer: association with complete response to therapy and improved survival. J Clin Oncol.

[25] Schulz U, Randalls B, Counsell C (2007). Anti-Hu syndrome: a rare presentation and a very difficult decision. Pract Neurol.

[26] Graus F, Keime-Guibert F, Rene R, Benyahia B, Ribalta T, Ascaso C, Escaramis G, Delattre JY (2001). Anti-Hu-associated paraneoplastic encephalomyelitis: analysis of 200 patients. Brain.

[27] Graus F, Abos J, Roquer J, Mazzara R, Pereira A (1990). Effect of plasmapheresis on serum and CSF autoantibody levels in CNS paraneoplastic syndromes. Neurology.

[28] Graus F, Vega F, Delattre JY, Bonaventura I, Rene R, Arbaiza D, Tolosa E (1992). Plasmapheresis and antineoplastic treatment in CNS paraneoplastic syndromes with antineuronal autoantibodies. Neurology.

[29] Sorhaug S, Steinshamn SL, Waldum HL (2005). Octreotide treatment for paraneoplastic intestinal pseudo-obstruction complicating SCLC. Lung Cancer.

[30] Vanek VW, Al-Salti M (1986). Acute pseudo-obstruction of the colon (Ogilvie’s syndrome). An analysis of 400 cases. Dis Colon Rectum.

[31] Coret F, Bosca I, Fratalia L, Perez-Griera J, Pascual A, Casanova B (2009). Long-lasting remission after rituximab treatment in a case of anti-Hu-associated sensory neuronopathy and gastric pseudoobstruction. J Neuro-Oncol.

